# Nosocomial Pathogens and Antimicrobial Resistance: Modern Challenges and Future Opportunities

**DOI:** 10.3390/microorganisms11071685

**Published:** 2023-06-28

**Authors:** Ana R. Freitas, Guido Werner

**Affiliations:** 1 1H-TOXRUN, One Health Toxicology Research Unit, University Institute of Health Sciences (IUCS), CESPU, CRL, 4585-116 Gandra, Portugal; 2 Laboratory of Microbiology, UCIBIO—Applied Molecular Biosciences Unit, Department of Biological Sciences, Faculty of Pharmacy, University of Porto, 4050-313 Porto, Portugal; 3 Associate Laboratory i4HB-Institute for Health and Bioeconomy, Faculty of Pharmacy, University of Porto, 4050-313 Porto, Portugal; 4 Division of Nosocomial Pathogens and Antibiotic Resistances, Department of Infectious Diseases, Robert Koch Institute, National Reference Centre for Staphylococci and Enterococci, Wernigerode Branch, 38855 Wernigerode, Germany

## 1. Introduction

Antimicrobial resistance (AMR) has become a critical global health emergency in the 21st century, with the greatest burden in resource-limited settings. The World Health Organization (WHO) has recognized AMR as one of the ten global public health challenges facing humanity, attributing the rise of drug-resistant pathogens primarily to the misuse and overuse of antimicrobials (https://www.who.int/news-room/fact-sheets/detail/antimicrobial-resistance (accessed on 26 June 2023)). A recent systematic analysis estimated 4.95 million deaths associated only with bacterial AMR in 2019, including 1.27 million deaths directly attributable to bacterial AMR, mostly caused by six leading pathogens (*Escherichia coli, Staphylococcus aureus, Klebsiella pneumoniae, Streptococcus pneumoniae, Acinetobacter baumannii*, and *Pseudomonas aeruginosa*) [1]. However, this burden estimation does not include other microorganisms such as fungi, which are increasingly being reported to be associated with severe and life-threatening human infections [2]. Indeed, in certain regions, the greatest concern regarding AMR lies with fungi, parasites (e.g., *Plasmodium falciparum*), and viruses (e.g., HIV), which are often overlooked in AMR surveillance initiatives. Fungi, in particular, have been largely neglected as a public health threat, despite the significant costs incurred by the healthcare sector due to antifungal resistance, exemplified by the emergence and global spread of human infections caused by multidrug-resistant *Candida auris* and azole-resistant *Aspergillus fumigatus*. In this context, the WHO recently launched a fungal priority pathogens’ list (WHO FPPL), ranking critical, high-, and medium-priority fungal pathogens, aiming to improve the overall response to the global rise of invasive fungal infections, particularly among immunocompromised populations [3]. The economic implications of AMR are substantial, extending beyond loss of life and disability, increasing healthcare costs and jeopardizing the effectiveness of medical interventions. Moreover, the threat of AMR extends beyond hospital settings, necessitating urgent collaborative efforts in an integrated One Health approach that encompasses all ecosystems. In March 2023, recognizing the urgency posed by health emergencies like AMR and food safety, the heads of the Quadripartite organizations (i.e., the World Health Organization (WHO), Food and Agriculture Organization of the United Nations (FAO), United Nations Environment Programme (UNEP), and the World Organisation for Animal Health (WOAH)) issued an unprecedented call for accelerated and prioritized global political action focusing on One Health as a unique solution to address future threats (https://www.who.int/news/item/27-03-2023-quadripartite-call-to-action-for-one-health-for-a-safer-world (accessed on 26 June 2023)). Finally, to effectively control the spread of AMR organisms and mitigate the burden of AMR-related diseases, there is a need for enhanced predictive intelligence to accurately assess the emergence of transmission of AMR within populations and healthcare settings. However, efforts to operationalize AMR forecasting in real time still face several constraints [4], and the implementation of efficient measures in different socioeconomic and geopolitical timeframes remains a major challenge [5].

This Special Issue of *Microorganisms* dedicated to nosocomial pathogens and AMR consists of a collection of nine articles addressing different aspects and the most recent developments of this topic. This issue presents recent studies investigating hospital outbreaks involving various multidrug-resistant pathogens such as vancomycin-resistant enterococci (VRE), methicillin-resistant *Staphylococcus aureus* (MRSA) and other staphylococci, multidrug-resistant Gram-negative bacteria (e.g., *Escherichia coli, Pseudomonas aeruginosa*), and *Candida auris* as an important fungal healthcare pathogen. Additionally, it covers epidemiological surveillance studies that explore the correlation between clinical *Enterococcus faecium*/MRSA populations and environmental sources. All studies employ a combination of epidemiological and genomic analyses to understand hospital outbreaks, transmission dynamics, resistance mechanisms, and the clinical implications of such events.

A thought-provoking review and opinion article summarizing the One Health dimension of AMR from a holistic, societal, and evolutionary perspective is provided by Coque et al. [5]. This opinion paper is divided into four sections: Reviewing socioeconomic factors contributing to the current healthcare system, reframing AMR in public and global health contexts, discussing the unit of analysis and indicators used in resistance surveillance, and addressing disparities, similarities, gaps, and challenges in combatting AMR. The study analyzes the complexities of hosts, microbes, and hospital environments, and their impact on surveillance, antimicrobial stewardship, and infection control. By integrating various aspects that are often approached individually and recognizing that AMR is a complex public health issue shaped by societal factors over centuries, Coque et al. highlight the lack of dialogue between stakeholders and the need for interdisciplinary collaboration and historical understanding to effectively solve existing “known unknowns” and address this complex public health issue.

A total of six articles are dedicated to leading hospital pathogens with substantial resistance progression and an immense strain level dynamic. The first study, by Bender et al. [6], reports on an outbreak of vancomycin-resistant *Enterococcus faecium* (VREfm) of unprecedented extent in southern Germany between October 2015 and November 2019. The outbreak affected a large number of patients (*n* > 2000), with most cases being colonization rather than (invasive) infections. In addition to its pure numerical dimensions, the immense strain dynamic over time is noticeable. The study highlights the importance of combining epidemiological and genomic analyses to comprehensively investigate outbreaks and emphasizes the need for timely implementation and strict execution of infection prevention and control measures to contain large VREfm outbreaks in hospital settings. 

Another study, by Correa-Martinez et al. [7], focuses on the molecular epidemiology of vancomycin-resistant enterococci (VRE) bloodstream infections (BSI) in the German federal state of North Rhine-Westphalia (NRW). The incidence of VRE BSI nearly tripled between 2016 and 2019, particularly in male patients aged 50 years and older. The study identifies the clonal lineage of MLST type ST117 as the predominant strain type associated with VRE BSI, which is also specified for other regions in Germany by studies published recently [8,9,10]. This paper highlights the benefits of genomic surveillance at the regional, federal, and national levels for the prevention of invasive infections caused by emerging VRE strains.

Despite decreasing trends in BSI throughout Europe, Methicillin-resistant *Staphylococcus aureus* (MRSA) remains an important healthcare-associated pathogen. In another paper of this Special Issue, by Silva et al. [11], MRSA strains from wastewater samples collected from three hospitals in Portugal were investigated. The MRSA isolates showed multidrug-resistant profiles and belonged to different genetic lineages, reflecting prevalent MRSA lineages like EMRSA-15 in the sampled healthcare institutions. The study highlights the presence of MRSA in hospital wastewater and underscores the possibility of sampling wastewater for surveying healthcare pathogens like MRSA.

*Staphylococcus lugdunensis* is a Gram-positive bacterium that belongs to the *Staphylococcus* genus and to the group of coagulase-negative staphylococci. *S. lugdunensis* has gained recognition as an important opportunistic pathogen responsible for a range of infections in humans. In a study by Fernández-Fernández et al. [12], *S. lugdunensis* isolates recovered from human samples in two Spanish hospitals were described and characterized. The isolates showed varying antimicrobial resistance profiles, antimicrobial activity production, and presence of the lugdunin bacteriocin gene. The findings highlight the potential pathogenicity of *S. lugdunensis* and its antimicrobial activity against coagulase-positive *Staphylococcus aureus*, emphasizing the need for further characterization and understanding of this emerging pathogen. 

Gram-negative, multidrug-resistant healthcare pathogens like carbapenem-resistant and carbapenemase-producing enterobacteria and non-fermenters are a focus of public health and medical interest worldwide. The present issue contains a study by Aguilar-Rodea et al. [13] describing the characterization of a *Pseudomonas aeruginosa* outbreak in a Mexican pediatric hospital using antibiotic susceptibility profiling, virulence factor production, and molecular typing. The outbreak isolates demonstrated extensive drug resistance and belonged to different strain types. The study highlights the importance of clinical, epidemiological, and molecular surveillance to finetune and streamline infection control measures to prevent the dissemination of high-risk *P. aeruginosa* strains in healthcare settings.

*Candida auris* is a fungal, healthcare-associated pathogen that has received substantial recent medical and public health attention. Infections caused by *C. auris* can spread in hospitals, particularly in ICUs. Antifungal resistance affects appropriate patient treatment and outbreak management. A case study by Corcione et al. [14] describes the colonization and infection of *C. auris* among critically ill patients in a transitional period between managing non-COVID-19 and COVID-19 patients in an Italian hospital. Eight patients were colonized and two developed invasive infections. The pathogen was cultured from various sites, with a median time of 24 days from admission to detection. Infection control measures, including rapid identification and isolation, remain crucial for treating and preventing infections in critically ill patients.

The two further articles in this issue focus on whole-genome-sequencing-based typing of mobile genetic elements in strains of two major superbugs. In the work by Freitas et al. [15], multidrug-resistant *E. faecium* strains causing human infections in Tunisian healthcare institutions were analyzed and compared to strains from diverse non-human sources. Genomic analysis revealed the presence of common multidrug-resistant *E. faecium* strains and large antibiotic resistance genetic elements carrying relevant adaptive traits across different hosts and regions. These findings underscore the importance of high-resolution genotyping in understanding the spread of multidrug-resistant strains within and between different sectors.

Infections caused by multidrug-resistant *E. coli* strains have increased, and plasmids play a major role in the spread of antibiotic resistance in *E. coli* and beyond all members of Enterobacterales. Tools for analyzing short-read sequencing data have been developed to predict plasmids, and in a study by Paganini et al. [16], 25 tools were reviewed, which were categorized into binary plasmid/chromosome classification and plasmid reconstruction tools. Of six benchmarked tools, MOB-suite was the most effective in reconstructing plasmids, including those carrying antibiotic resistance genes (ARGs). However, most tools struggled to completely recover ARG-carrying plasmids. The findings serve as a guideline for selecting appropriate plasmid reconstruction tools when studying *E. coli* plasmids without long-read sequencing data.

In conclusion, this Special Issue features nine captivating articles that delve into cutting-edge aspects of nosocomial pathogens and antimicrobial resistance. The studies included in this issue offer valuable recent insights into hospital outbreaks, dissemination events, and advanced typing methods related to critical multidrug-resistant pathogens, shedding light on their transmission dynamics, resistance mechanisms, and clinical implications. Together, these articles raise awareness about the challenges we face in maintaining effective healthcare practices and emphasize the need for enhanced One Health surveillance and infection control measures.

## Data Availability

Not applicable.
